# Are flat feet a disadvantage in performing unilateral and bilateral explosive power and dynamic balance tests in boys? A school-based study

**DOI:** 10.1186/s12891-023-06752-9

**Published:** 2023-07-31

**Authors:** Peter Sagat, Peter Bartik, Lovro Štefan, Vangelis Chatzilelekas

**Affiliations:** 1 GSD/Health and Physical Education Department, Sport Sciences and Diagnostics Research Group, Prince Sultan University, Riyadh, 11586 Saudi Arabia; 2 Department of Physical Activities and Health Sciences, Faculty of Sports Studies, Masaryk University, Brno, 625 00 Czech Republic; 3 Sports center for disabled persons, Rodos, Greece

**Keywords:** Flat feet, Children, Physical performance, Differences, Explosive power, Speed, Balance

## Abstract

**Background:**

Little evidence has been provided regarding physical performance and flat-footedness in school-age children. Although flat feet may decrease the level of motor performance, findings remain inconsistent. Therefore, the main purpose of the study was to determine whether children with flat feet had poorer physical task performance, compared to normal-footed children.

**Methods:**

A total of 208 primary school boys were included in the study (107 normal-footed and 101 flatfooted boys). Flat footedness (< 42°) was determined using Clark’s method. The children were tested by a set of unilateral and bilateral tests selected from the area of ​​explosive power and dynamic balance which included: (i) countermovement jump, (ii) standing broad jump, (iii) the triple crossover hop for distance test, (iv) maximal sprinting speed over 10, 20 and 40 m and (v) the Star Excursion Balance Test. Differences were adjusted for age, body mass index, peak height velocity and physical activity.

**Results:**

Flat footed children exhibited significantly poorer results in bilateral standing broad jump (effect size [ES] = 0.34), unilateral standing broad jump for dominant (ES = 0.31) and non-dominant leg (ES = 0.20), the triple crossover hop for distance test for dominant (ES = 0.24) and non-dominant leg (ES = 0.23) and the Star Excursion Balance Test (ES = 0.23–0.43) and were slower in maximal sprinting speed test over 20 m (ES = 0.25) and 40 m (ES = 0.30).

**Conclusions:**

This study shows that children with flat feet performed poorer in some physical performance tasks, compared to the normal feet counterparts.

## Background

Flatfoot is a medical condition associated with the absence or lowered medial longitudinal arch [1]. Although flat feet represent a clinical concern for parents, intervention guidelines for children with flat feet remain unknown [2]. Despite that, flat feet are constantly being treated with arch supports, corrective shoes and inserts [3]. Estimates suggest that the prevalence of flat feet in primary school children is between 15% and 25% with a decreasing trend with age [1, 4, 5]. Definition of flatfoot in children is still confusing and general classification differentiates between physiological and pathological causes [6]. Physiological flatfoot is often natural and comes with development, but can be more pronounced in overweight and obese children [1, 4–6]. On the other hand, pathological flatfoot may be responsible for pain and discomfort, which significantly manifests in adulthood and leads to poorer physical performance [7, 8].

Physical performance has been considered a useful and powerful marker of health in children [9]. The foot represents the first contact with the ground, and if such structure is weak or damaged, it may impact physical performance. With that in line, previous evidence has shown that deviated foot structure (meaning low or high arch of the feet) can result from the transfer of foot eversion to internal rotation of the tibia while running [10, 11]. However, there has been a lack of studies exploring the associations between flat foot and both explosive and dynamic balance performances. Available literature has suggested that flatter feet may be related to a muscle deficiency [12], which can indirectly affect physical performance. Conversely, other studies did not confirm that flat footedness was associated with physical performance, even after comparing children with very low and children with very high arches [13]. Despite a relatively high prevalence of flat footedness in children, not many attempts have been made to establish whether normal-footed children may perform better in physical performance tests.

Therefore, the main purpose of the study was to determine if children with flat feet might have poorer physical performance, compared to children with ‘normal’ feet. We hypothesized that flat-footed children might exhibit lower physical performance values.

## Methods

### Study participants, design and procedure

**Fig. 1:**
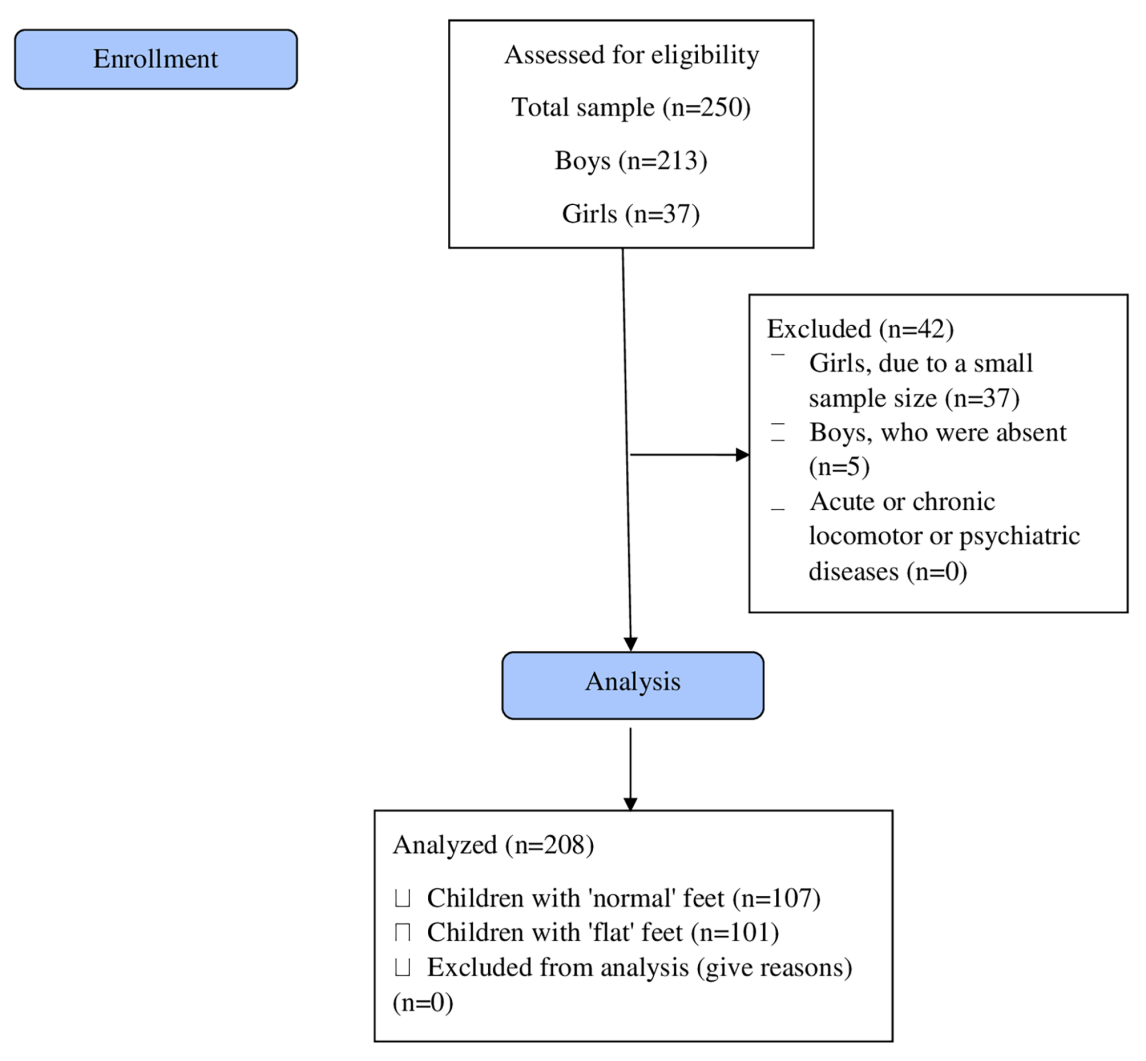
A flow chart diagram for the recruitment of the participants

In this cross-sectional study, we randomly selected 10 out of ≈ 180 primary schools in the city of Zagreb. Out of 10 schools, 5 of them agreed to participate. Within each school, we randomly selected one class representing one age category (children between age 12 and 14). This would give a total of 10 classes with ≈ students. After collecting an informed consent for participation, our sample mainly consisted of boys (85%). Thus, the analyses were based on primary school-aged boys. By using sample size analysis with statistical power of 0.80, α < 0.05, effect size of 0.25 and the allocation ratio of 1, the appropriate total sample size to detect significant differences would be 156. Due to a possible drop-out rate, we strengthened our sample to consist of 208 primary school boys aged 12 to 14 [mean (SD) age: 13.0 ± 0.6 y; height: 168.3 ± 9.3 cm; weight: 60.9 ± 15.1 kg; body mass index: 21.3 ± 4.0 kg/m^2^). The criteria for inclusion in the study were physically active and healthy students who regularly attended physical health education classes and being without significant deformities of locomotor system, while the exclusion criteria consisted of children who did not attend physical education classes on regular basis or suffered from acute/chronic locomotor or psychiatric diseases at the time the study had been conducted (a flow chart diagram in Fig. 1). The participants were not familiar with the objectives of the study, and their participation was confirmed by their parents. Only participants with physiological or flexible flat feet were included in the research, while subjects with a fixed or rigid flat foot were excluded. The research was conducted with the ethical code Council for children as an advisory body of the Government of the Republic of Croatia and with approval Committees for scientific work and ethics of the Faculty of Kinesiology, University of Zagreb (ethical code: 03/2016). All methods were carried out in accordance with relevant guidelines and regulations of the Declaration of Helsinki. The written informed consent was voluntarily signed by the participants, participants’ parents or their guardians to have data from their records used in research.

### Flat feet assessment

The podiatric examination performed by an experienced podologist was done with a podoscope. Each participant stood barefoot with both feet on the podoscope glass, while standing still with weight being equally distributed on both legs, shoulders relaxed, legs slightly hip width apart, heels slightly parallel, and head placed straight forward. Podoscope was connected to a camera that was switched on and connected to the laptop, and thus recorded both feet and archived them in a special software program (Video Pack–videography). The diagnosis of foot function was evaluated using a podoscope in a static examination. Flat footedness was evaluated using Clark’s method, a practical, reliable and sensitive metric for quantification of medial arch height in children and recommended for research and clinical practice [14]. As described in the literature, the Clark’s method is based on calculating Clark’s angle, which is defined as ‘the angle between the tangent at the medial margin of the footprint and the line connecting the longest perpendicular distance from the medial border of the foot and the point at which the medial tangent crosses the margin of the front foot’. When the Clark’s angle is calculated, foot posture was dichotomized as ‘normal’ (Clark’s angle, ≥ 42º) or ‘flatfoot’ (Clark’s angle, < 42º) arch [14–16].

### Physical performance assessment

The area of ​​motor skills was covered by a set of tests selected from the area of ​​explosive power and dynamic balance. To determine the explosive abilities of children, variables to assess the speed-powerful properties of the muscles, vertical and horizontal jumping performance were used.

Eccentric-concentric countermovement jump was used to estimate the vertical component of explosive power. The task was performed three times with the best score being recorded as final in centimeters (cm). A single jump started with straight legs performing a natural flexion before the takeoff phase with hands held at the hips during performance. The test was repeated for both bilateral (both legs) and unilateral (dominant vs. non-dominant leg) condition and the rest interval between the trials was set at 3 min.

Standing broad jump assessed the level of horizontal component of explosive power. Each participant was instructed to perform a distance jump from a standing start while bending their knees with their arms in front of them. The feet were parallel to the ground and when ready, they swung both arms and jumped forward vigorously as far as possible, trying to land with their feet together while being in an upright position. The task was performed three times with a 1-min rest interval and the best score was taken as the final score in cm. The same procedure was applied for the countermovement jump, where all participants performed standing broad jump in bilateral and unilateral condition.

The triple crossover hop for distance test estimated unilateral explosive power [17]. Participants had to perform three consecutive hops for maximum distance in a forward direction (all on the same limb, and without pausing in between each hop except for the final landing), though crossing back and forth over a custom-made 15 cm width mat, without touching the mat. The trial was considered successful if the participant landed in a controlled manner on the final hop. The test for each leg was performed three times with a 1-min rest interval and the best score was recorded in cm.

Maximal sprinting speed was tested over 10, 20 and 40 m. Times in milliseconds (ms) at each point were recorded by eight infra-red timing gates (Fusion Sport Smart Speed, Fusion Sport, 2 Henley ST Coopers Plains, QLD, 4108, Australia) positioned at the start and at 10, 20 and 40 m. The participant held their starting position by putting their lead foot on a line 15 cm behind the first timing gate. The time was recorded from when the participant intercepted the first timing gate. The test was performed three times with 5 min rest intervals between each trial.

Dynamic postural control balance was evaluated by the Star Excursion Balance Test, a rehabilitative tool that uses a series of single-limb squats to reach maximally in order to touch a point along 1 of 8 designated lines on the ground arranged in a grid that extends from a center point and which are 45° from one another [18, 19]. Each line represents one direction and are named to the stance limb as anterior, anteromedial, anterolateral, medial, lateral, posterior, posteromedial and posterolateral. The purpose of the test is to reach as far as possible along each reaching line and to lightly touch the line with the most distal part of the foot, while standing steadily on a single limb. When the first part of the task is completed, the reaching limb returns to the beginning position in the center without losing balance control [18, 19]. If we observed that the participant touched heavily, came to rest when performing the task or maintained balance when lifting and shifting any part of the foot, the trial was discarded and repeated. The task was done 3 times in each direction and the best score was used as final in cm.

Previous studies have shown that age [13], body mass index [4, 5], peak height velocity [20] and physical activity [21] may mediate the association between flat footedness and physical performance, which we included in adjusted models. Age was self-reported. Height and weight were objectively measured using Seca stadiometer and digital scale with a precision of 0.1 mm and 0.1 kg. Body mass index was calculated with the following formula: [body mass index = weight (kg)/height (m)^2^]. Peak height velocity was based on the equation proposed by Mirwald et al. [22]., which used age, leg length, sitting height, height and weight to predict maturity offset in boys. The level of physical activity was assessed using Physical Activity Questionnaire for Older Children (PAQ-C). The PAQ-C is a self-administrated, 7-day recall instrument with 10 questions regarding the level of physical activity in: (i) spare time, (ii) during physical education, (iii) during breaks between classes, (iv) during lunch breaks, (v) right after school, (vi) during evenings, (vii) during last weekend, (viii) self-evaluated and (ix) for each day last week. Each question was scored from 1 (the lowest activity response) to 5 (the highest activity response), and the mean of all 9 questions was taken to create the total physical activity score [23].

### Testing protocol

The measurements were carried out in the morning hours, during the physical and health education classes in the sports halls. The measurement protocol in all schools was the same, and the measurements were carried out by the same group of researchers to avoid measurement error. Each researcher (measurer) performed the same type of measurement in all schools. Before starting the measurements, the subjects underwent a standardized warm-up protocol, which consisted of a 10-minute run with tasks and stretching exercises for the muscles of the lower extremities. In order to obtain a more detailed insight into the foot function of the dominant and non-dominant foot, it was necessary to use a larger number of tasks to assess different motor abilities and related properties based on anthropometric measurements.

### Statistical analyses

Basic descriptive statistics are presented as mean and standard deviation (SD). To examine differences between ‘normal’ vs. ‘flat’ foot group of children, we used the analysis of covariance (ANCOVA) adjusted for age, body mass index, peak height velocity and the level of physical activity. The magnitude of the differences between the groups in each variable was calculated using Cohen’s D effect size (ES) with 95% confidence interval (95% CI). According to Hopkins et al. [24]., ES was classified as trivial (< 0.2), small (0.2–0.6), moderate (0.6–1.2), large (1.2–2.0), very large (> 2.0) and extremely large (> 4.0). In addition, to detect differences of multiple physical performance tests, we performed the Benjamini-Hochberg Procedure, which decreases the false discovery rate [25]. In practical sense, the analysis adjusts the rate and helps to avoid Type 1 errors (false positives). A preliminary analysis showed that out of 27 *p*-values, 7 of them were different than in initial analyses, but significant *p*-values that we obtained for physical performance tests remained significant. All statistical analyses were conducted using Statistical Packages for Social Sciences version 23 (SPSS Inc., Chicago, IL, USA). Two-sided p-values were used, and significance was set at α < 0.05.

## Results

Basic descriptive statistics are presented in Table 1. Of total sample, 48.6% of boys were diagnosed with flat feet (*N* = 101), compared to children with ‘normal’ feet (*N* = 107). Although no significant differences in demographic variables were observed, children with flat feet were heavier and had greater body mass index values, compared to children with ‘normal’ feet.

**Table 1:** Basic descriptive statistics of the study participants (*N* = 208)

Study variables	Total (*N* = 208)	‘Normal’ footed children (*N* = 107)	‘Flat’ footed children feet (*N* = 101)	Cohen’s D (95% CI)	*p*-value
Age (y)	13.6 (0.6)	13.0 (0.6)	13.6 (0.6)	0.01 (-1.3–0.7)	0.723
Height (cm)	168.3 (9.3)	167.8 (9.1)	168.9 (9.6)	-0.12 (-0,39–0,15)	0.396
Weight (kg)	60.9 (15.1)	59.1 (14.4)	62.7 (15.7)	-0.24 (-0,56–0,14)	0.087
Body mass index (kg/m^2^)	21.3 (4.0)	20.8 (3.8)	21.8 (4.2)	-0.25 (-0.52–0.02)	0.080
Peak height velocity	-1.5 (0.6)	-1.5 (0.6)	-1.4 (0.6)	0.17 (-0.44–0.11)	0.588
Physical activity (score)	3.1 (1.2)	3.2 (1.3)	3.0 (1.2)	0.16 (-0,11–0.43)	0.303

*P* < 0.05

**Fig. 2:**
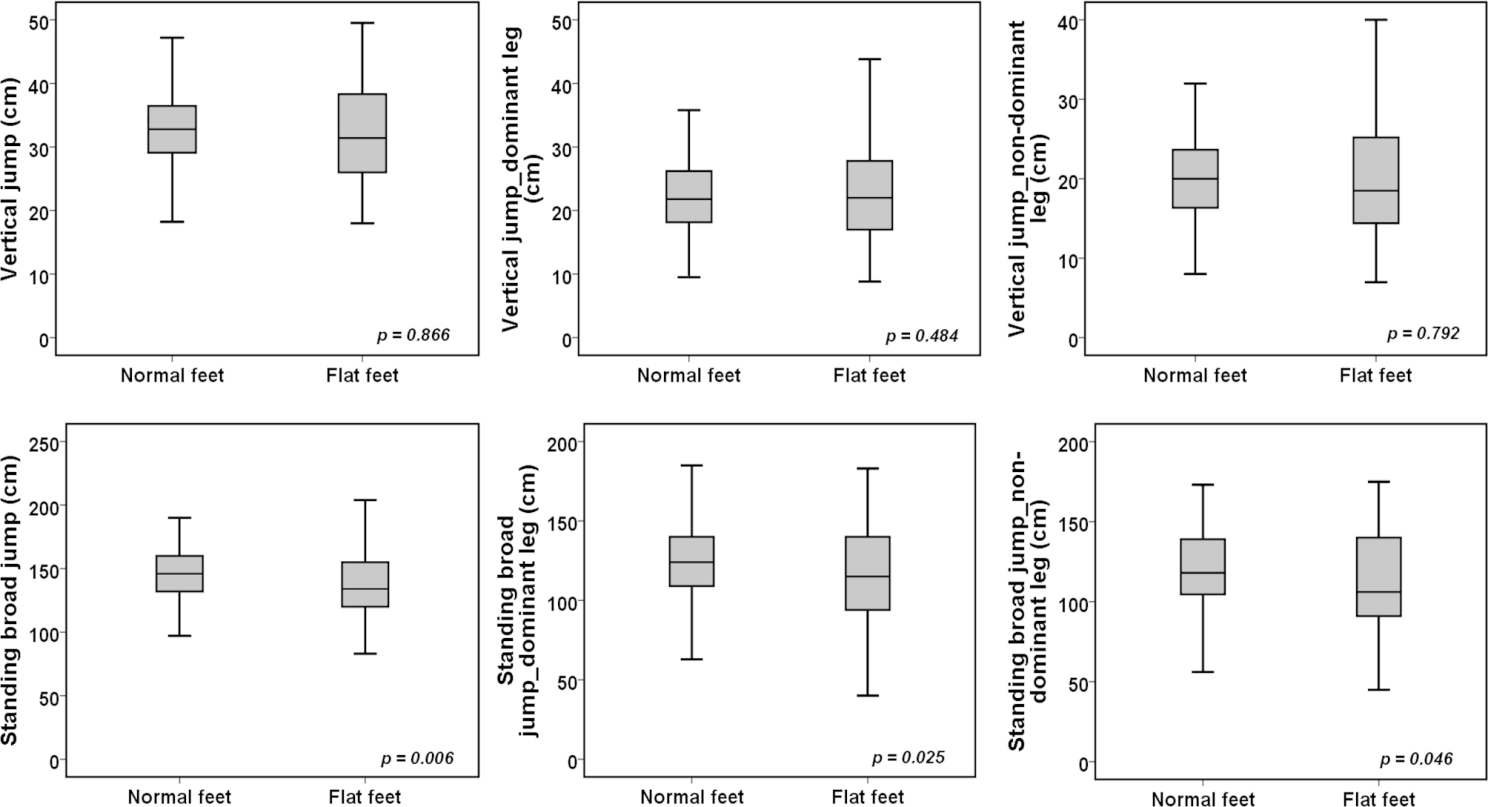
Differences in unilateral and bilateral vertical and horizontal jumps between ‘normal-footed’ and ‘flat-footed’ boys

**Fig. 3:**
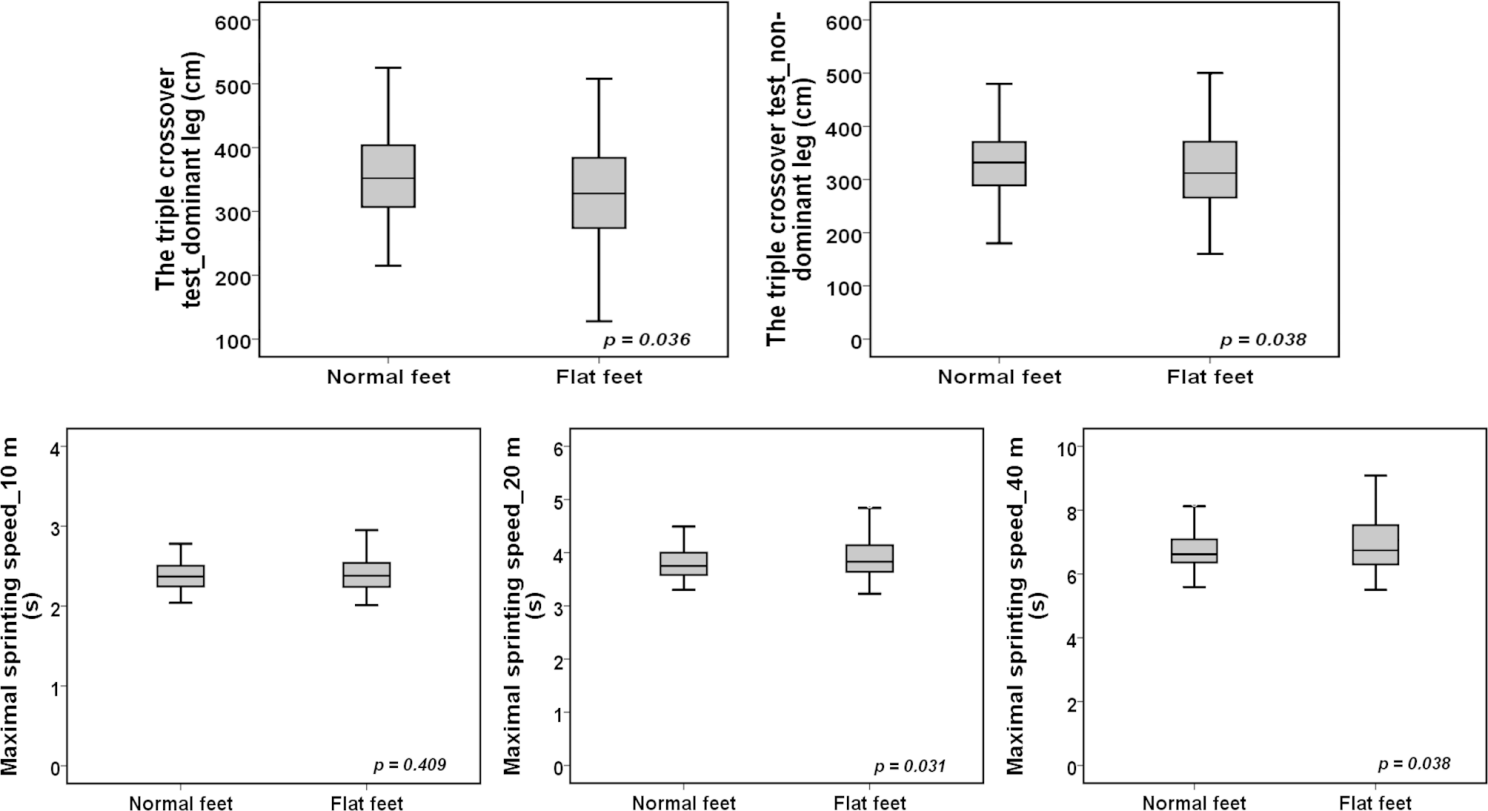
Differences in the triple crossover hop for distance test for dominant and non-dominant leg and maximal sprinting speed over 10, 20 and 40 m between ‘normal-footed’ and ‘flat-footed’ boys

**Fig. 4:**
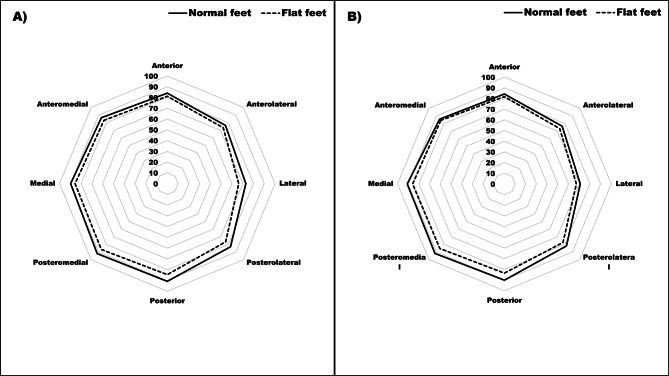
Differences in the Star Excursion Balance Test for dominant (**A**) and non-dominant (**B**) leg between ‘normal-footed’ and ‘flat-footed’ boys

Table 2 shows the differences between the flat-footed and normal-footed children adjusted for age, body mass index, peak height velocity and level of physical activity. In general, flat-footed children exhibited significantly poorer results in bilateral standing broad jump to a small extent (ES = 0.34), standing broad jump performed with dominant leg to a small extent (ES = 0.31) and standing broad jump performed with non-dominant leg to a trivial extent (ES = 0.20) (Fig. 2). The mean performance in triple jump with dominant and non-dominant leg was significantly poorer to a small extent (ES = 0.24 and ES = 0.23) in flat-footed than in normal-footed children (Fig. 3). Small significant mean differences were observed in maximal sprinting speed over 20 m (ES = 0.25) and 40 m (ES = 0.30) between flat-footed and normal-footed children in favor of normal-footed children (Fig. 3). Finally, flat-footed children performed poorer to a medium extent in dynamic postural balance performed in posteromedial (ES = 0.36), posterior (ES = 0.43), posterolateral (ES = 0.42) and lateral (ES = 0.40) directions for dominant leg and in posteromedial (ES = 0.41), posterior (ES = 0.41) and posterolateral (ES = 0.23) directions for non-dominant leg (Fig. 4).

**Table 2:** Differences in physical performance tests between flatfooted and normal-footed children

Physical performance tests	‘Normal’ footed children (*N* = 107)	‘Flat’ footed children feet (*N* = 101)	Mean difference (95% CI)	Cohen’s D (95% CI)	*F*-value (*p*-value; partial eta^2^)*
	Mean ± SD	Mean ± SD			
**Vertical jump (cm)**					
Bilateral	33.7 ± 6.4	33.9 ± 8.1	-0.17 (-2.17 to 1.83)	-0.03 (-0.30 to 0.24)	2.590 (0.077; 0.025)
Dominant leg	22.0 ± 5.6	22.6 ± 7.2	-0.62 (-2.38 to 1.13)	-0.09 (-0.37 to 0.18)	0.769 (0.465; 0.007)
Non-dominant leg	20.1 ± 5.3	19.9 ± 6.8	0.22 (-1.45 to 1.89)	0.03 (-0.24 to 0.30)	0.689 (0.503; 0.007)
**Standing broad jump (cm)**					
Bilateral	145.0 ± 22.5	136.8 ± 25.5	8.11 (1.54 to 14.48)	0.34 (0.07 to 0.61)	5.842 (0.003; 0.054)
Dominant leg	123.7 ± 25.0	115.4 ± 27.8	8.35 (1.12 to 15.58)	0.31 (0.04 to 0.59)	4.072 (0.018; 0.038)
Non-dominant leg	117.4 ± 24.6	112.1 ± 28.6	5.34 (0.94 to 12.61)	0.20 (0.07 to 0.47)	3.211 (0.042; 0.030)
**Triple jump (cm)**					
Dominant leg	350.5 ± 64.0	334.3 ± 68.5	16.10 (0.43 to 35.64)	0.24 (0.03 to 0.52)	2.129 (0.049; 0.020)
Non-dominant leg	333.0 ± 66.7	317.3 ± 70.5	15.70 (0.14 to 35.58)	0.23 (0.04 to 0.51)	2.247 (0.046; 0.022)
**Sprint (s)**					
10 m	2.37 ± 0.23	2.40 ± 0.25	-0.04 (0.10 to 0.03)	-0.13 (-0.40 to 0.15)	0.151 (0.860; 0.001)
20 m	3.82 ± 0.37	3.92 ± 0.42	-0.10 (-0.21 to 0.00)	0.25 (0.02 to 0.53)	2.533 (0.038; 0.025)
40 m	6.72 ± 0.71	6.96 ± 0.88	-0.24 (-0.46 to -0.03)	0.30 (0.03 to 0.57)	4.236 (0.010; 0.040)
**Dynamic balance_dominant leg (cm)**					
Anterolateral	75.4 ± 8.5	74.4 ± 9.9	1.01 (-1.52 to 3.54)	0.11 (-0.16 to 0.38)	2.971 (0.053; 0.028)
Anterior	83.0 ± 8.6	82.1 ± 9.3	0.88 (-1.57 to 3.33)	0.10 (-0.17 to 0.37)	2.182 (0.067; 0.019)
Anteromedial	85.5 ± 9.4	84.1 ± 9.3	1.40 (-1.16 to 3.95)	0.15 (-0.12 to 0.42)	2.118 (0.116; 0.021)
Medial	81.5 ± 10.0	79.6 ± 10.7	1.89 (-0.94 to 4.73)	0.18 (-0.09 to 0.46)	1.760 (0.175; 0.017)
Posteromedial	84.1 ± 9.8	80.5 ± 10.4	3.63 (0.87 to 6.40)	0.36 (0.08 to 0.63)	4.297 (0.015; 0.042)
Posterior	83.5 ± 10.2	78.8 ± 11.8	4.65 (1.65 to 7.66)	0.43 (0.15 to 0.70)	3.970 (0.020; 0.037)
Posterolateral	76.1 ± 10.9	71.3 ± 12.2	4.78 (1.62 to 7.93)	0.42 (0.14 to 0.69)	3.193 (0.043; 0.030)
Lateral	66.1 ± 11.2	61.3 ± 12.6	4.77 (1.50 to 8.03)	0.40 (0.13 to 0.68)	3.439 (0.040; 0.031)
**Dynamic balance_non-dominant leg (cm)**					
Anterolateral	75.4 ± 8.7	74.2 ± 9.4	1.17 (-1.30 to 3.64)	0.13 (-0.14 to 0.40)	1.091 (0.338; 0.011)
Anterior	83.2 ± 8.7	82.7 ± 9.1	0.53 (-1.90 to 2.96)	0.06 (-0.22 to 0.33)	2.984 (0.053; 0.028)
Anteromedial	84.8 ± 8.3	85.2 ± 10.6	-0.15 (-2.53 to 2.23)	0.04 (-0.31 to 0.23)	2.718 (0.056; 0.025)
Medial	88.9 ± 10.5	86.9 ± 11.6	2.06 (-0.96 to 5.08)	0.18 (-0.09 to 0.45)	0.899 (0.409; 0.009)
Posteromedial	91.0 ± 10.1	86.5 ± 11.6	4.51 (1.54 to 7.48)	0.41 (0.13 to 0.68)	4.200 (0.016; 0.039)
Posterior	89.1 ± 10.5	84.5 ± 12.2	4.62 (1.50 to 7.73)	0.41 (0.13 to 0.68)	3.208 (0.042; 0.030)
Posterolateral	81.1 ± 10.5	78.5 ± 12.2	2.59 (0.52 to 5.71)	0.23 (0.04 to 0.50)	2.991 (0.050; 0.029)
Lateral	70.0 ± 12.1	68.3 ± 12.8	1.70 (-1.70 to 5.11)	0.14 (-0.14 to 0.41)	0.290 (0.748; 0.003)

*model adjusted for age, body mass index, peak height velocity and the level of physical activity

*P* < 0.05

## Discussion

The main purpose of the study was to determine whether children with flat feet exhibit poorer results in physical task performance, compared to children with ‘normal’ feet. The main findings are: (i) boys with ‘flatter’ feet perform significantly worse in bilateral and unilateral standing broad jump tests, the triple crossover hop for distance test and in the maximal sprinting speed over 20 and 40 m test; (ii) boys with flat footedness also exhibited poorer results in the Star Excursion Balance Test for posteromedial, posterior, posterolateral and lateral directions.

Our results are in line with previously published evidence [7]. In a study by Lin et al. [7]., the authors investigated the associations between low-arched feet and performance skills and found that flatfooted children scored lower than children without flat footedness. In brief, a series of tasks, including squatting and standing up on toe without support, toe- and heel-walking, one-leg hopping, and standing were performed and children with ‘normal’ feet or ‘mild’ flat feet had better results, compared to their counterparts with ‘moderate’ and ‘severe’ flat feet [7]. On the contrary, Tudor et al. [13]. showed that flat footedness is not a limiting factor for task performance, where physical performance was similar and did not depend on foot morphology and function. Conflicting results may be due to different methodology of assessing flat footedness and physical fitness tests to evaluate performance. For example, a study by Tudor et al. [13]. used different tests and age ranges, compared to our study. The same group of authors also corrected arch height index by age, to create arch index residuals. However, we found no significant correlation between age and Clark’s angle. Similar findings were obtained by the study of Lin et al. [7]., where they used subjective method of visualization to determine flat footedness in preschool children, while we used more objective assessment of arch height measured by a podoscope and Clark’s method. Also, a higher prevalence of flat footedness (57%) was found in their study [7], where most physiological and developmental foot changes start to occur in the first decade of life [26]. Unfortunately, we were not able to test the number of injuries, which mediate the association between foot morphology and physical performance. On the other hand, several previous studies have shown that flatter feet may play a protective role against overuse injuries, compared to individuals with ‘larger’ arch height [15]. Thus, it is still very difficult to suggest and confirm, whether flat feet should be under treatment, because there is a general lack of objective criteria to assess functional deviations in a kinetic chain [13]. We also showed a greater variance in Fig. 1 for flatfooted children, compared to their normal-footed counterparts. According to aforementioned mechanisms, it is possible that flatfooted children have previously suffered from higher incidence of foot injuries. Unfortunately, we were unable to collect the data regarding foot-specific injuries. On the other hand, previous evidence suggests that flatfooted children may have greater weight and body mass index values than their normal-footed peers, which may influence the performance. Whereas we did not find significant differences in weight and body mass index values between flatfooted and normal-footed children, flatfooted children in our study were heavier and had higher body mass index values.

Despite the high prevalence of flat footedness in preschool and primary school children, there has been conflicting evidence to confirm the general opinion and speculation that flat feet may be responsible for poorer physical performance. Although previous studies have claimed that treatments for flat footedness are not effective [27], we still found marked differences between children with ‘normal’ vs. ‘flat’ feet in horizonal explosive power tasks, maximal sprinting abilities and dynamic balance, especially in postural direction. A decrease in some tasks of physical performance in this study may be explained by poor postural stability, foot pathologies, pain and discomfort in flat footed children [27]. Since we did not collect additional information regarding foot function and the prevalence of injuries, we could only speculate that flat feet led to markedly lower results in some performance tests. Biomechanical studies combining kinematic and kinetic gait parameters have shown that flat footedness may also result in different rotational forces that dominate in the lower leg and slower muscle activation [11].

This study is not without limitations. First, by using a cross-sectional design, we cannot determine the causal association between flat feet and physical performance. It is possible that children with lower levels of physical activity may suffer from overweight/obesity, which in addition may lead to flatter feet [21]. Second, we only included boys in our study, and our results must be interpreted with caution. Third, we did not collect information about the regular type of shoes worn or the prevalence and the type of injuries. Fourth, this study only included boys aged 12 to 14 from one city in Croatia, which may limit the generalizability of the findings to other populations. Additionally, while the study aimed to minimize measurement error by using the same protocol and researchers, there is still the potential for variability in the measurements due to factors such as differences in foot positioning or pressure during the assessments. Therefore, future research should be prospectively conducted on a larger sample of schoolchildren, in order to investigate causal associations between foot structure and function and physical performance.

## Conclusion

In summary, we found that children with ‘flat’ feet might exhibit poorer results in physical performance tests, especially in bilateral and unilateral horizontal explosive power, maximal sprinting speed and postural direction of dynamic balance in 12 to 14-year-old boys. Observed differences in this sample ranged from trivial to medium; therefore, further research is needed to clarify the flat-footedness influence on performance measures in school-aged children. Although the controversy about flat footedness is still ongoing, our study is just a contribution to the literature of exploring the differences between children with ‘flat’ vs. ‘normal’ feet in physical performance tests.

## Data Availability

The datasets used and/or analyzed during the current study are available from the corresponding author on reasonable request.

## List of Abbreviations

ANOVA: Analysis of variance
ES: Effect size
PAQ: C - Physical Activity Questionnaire for Older Children
SD: Standard deviation
95% CI: 95% confident interval
